# Treatment of Alveolar Abscess

**Published:** 1855-01

**Authors:** Charles W. Ballard


### Selected Articles.

From the New York Medical Gazette.

### TREATMENT OF ALVEOLAR ABSCESS.

BY CHARLES W. BALLARD, M. D., D. S. S.

The following method of treating Alveolar Abscess has not, I believe, been laid before the Profession. I have been pursuing it for several years with an almost uniform success, and now feel warranted in recommending the practice. I do not think the number of failures to cure abscess of from ten days' to two years' standing, have amounted to five per cent. on the number treated. It must be understood, however, that I have by this method treated no case where the disease has been caused by external violence, applied so as to injure the alveolar processes, and but few where it has been the result of spontaneous death of the nervous pulp without apparent exposure or injury from the surface; in these few, however, the success of the treatment has been of the most satisfactory nature.

Almost all cases of alveolar abscess are caused by the decomposition of the pulp, which, etxending to, or in its results affecting the periosteum of the tooth, gives rise to a greater or less degree of inflammation of that membrane; lymph is thrown out, a sack is formed, which, becoming filled with pus, eventually bursts, and its contents force an opening through the gum, causing the unsightly and often painful abscess, and from which is discharged a quantity of matter of the most injurious and often offensive character.

It is obvious that the exciting and continuing cause of this discharge must be the decomposed substance contained in the nerve cavity of the tooth. Of course no plan of treatment could prove suecessful which did not entirely eradicate the decomposed mass; and experience has taught me that unless every *trace* of decomposition in or about the tooth is removed, no permanent success can be relied upon or even expected. I had been led to believe, that all that could be done in these cases, was to remove the decay from the tooth, as well as the decomposed substance contained in the nerve cavity, and then, after filling the hole with gold precisely as though the nerve had just been excised and removed, and no abscess had ever existed, to cauterize in one of the various ways the abscess or external opening upon the gum; and my impression is, that at the present time this course of procedure constitutes the main reliance of the Dental profession for the cure of this most annoying disease.

After operating in this manner for some time, and finding my success not equal to expectation, I set it down as a poor sort of "forlorn hope" operation, not to be brought into requisition until other treatment had failed, and not to be depended upon even then. In theory the operation seemed good, but there was a link wanting to enchain success to the practice, and this link, I believe, I have supplied.

Some three years since, a patient came to me complaining of an abscess that had become almost unbearable. The history she gave of it was an "oft-repeated tale." The tooth—a superior left canine—had become diseased; in the attempt to remove the carious portion, so much pain was experienced, that the operator determined to put something (arsenic, I suppose,) into it, in order to remove the sensibility. After a few days, the decay was removed and the tooth filled. In a few weeks it began to give trouble; to ache; to feel a little loose, and to seem a little longer than the other teeth. Soon it commenced to throb, the face to swell, and then an abscess formed and discharged, relieving all the previous symptoms, but continuing, up to the time of application to me, to discharge a fluid, which, becoming more and more offensive, had, at the end of two years, rendered the patient's situation an exceedingly unpleasant one; the most mortifying part of which was the consciousness that the diseased tooth caused her to be an annoyance to her friends. Although I had often met with similar cases, I had never attempted to cure an abscess of so long standing.

The patient declared her willingness to have the tooth extracted, provided no other mode of relief could be suggested. After explaining to her the method of operating pursued in such cases, and informing her of the poor chance of success, she requested me to experiment with a view to both save the tooth and rid her of her trouble. Upon removing the filling and cutting through the nerve cavity, with the intention of removing the remains of the decomposed nerve, I was much astonished at finding the cavity *empty, and perfectly clean and dry*, and yet connected with this tooth was an alveolar abscess of the very worst description, and which I had every reason to believe originated in the nerve cavity. This cavity had never been opened before, so that it was impossible for the nerve to have been removed by manipulation. According to the generally received opinion, this abscess "had no business to be an abscess;" it had no valid excuse for existence. There were no remains of nerves or decomposed matter visible; the canal was perfectly dry; upon filling it to the foramen with floss silk, and then withdrawing the strands, no moisture or discoloration could be detected upon them. In fact, there were but two reasons why the case could not have been taken for a recently performed operation of removal or extirpation of the nerve: one of these was the absence of that peculiar pinkish shade always noticed in the parietes of the nerve cavity, and which is due to the translucent properties of healthy dentine; and the other, the exhalation of the odor mentioned above, which was by many degrees more powerful than pleasant. Not caring to proceed hastily in this case, and having full license from the patient to experiment, I dismissed her after having made another appointment.

I freely confessed that this case perplexed me exceedingly. After studying it for some time, I came to the conclusion that the decomposed nerve had been *mostly* got rid of by means of the abscess; that the remainder had been infiltrated into the dentine, and that the effluvia arising from this portion being exceedingly acrid, had caused sufficient irritation to prevent the abscess from closing. I considered the trifling discharge from the abscess to proceed from the sack at the apex of the root, a result of the acrid nature of the odor proceeding from the tooth, and that this last was caused by the decomposed animal matter contained in the dentinal tubes.

Upon removing the dentine from the sides of the cavity, which I did upon the return of the patient, and finding that I gained little or nothing by so doing, I determined, for my own comfort, as well as for the purpose of ascertaining the value of the antiseptic powers of the drug, to substitute the vapor of creosote for the one so noxious. This I did, closing the cavity air-tight, with wax immediately after introducing the creosote.

The next time I saw my patient she remarked that there had been a marked improvement, but that traces of creosote had been noticed. It was at once proved that the diag nosis had been a correct one—the vapor of the drug having forced its way through the canal, foramen, and abscess, into the mouth, and, of course, tainting the breath. The advantage gained by this experiment was so manifest, that I determined to renew it; This I did several times, and then filling the cavity with floss silk, and closing the opening, I requested the patient to call at the end of the week.

At the appointed time I found, upon examination, that all traces of creosote had disappeared, and that the former trouble remained in a greatly reduced degree. I recommenced the treatment with creosote, and after another respite of a week (the tooth meanwhile being filled with floss silk only,) I found that there was but a slight trace of creosote, and none at all of the peculiar odor which had been the cause of so much annoyance. A few days after this, I filled both crown and fang with gold, and in two weeks from that time, without any further treatment, the abscess had entirely disappeared. The success was certainly encouraging, and after repeating the experiment, in a number of instances with a like result, I was induced to adopt a similar mode of treatment for all cases of alveolar abscess to which it could be applied, occasionally making slight modifications to suit individual peculiarities.

My usual method of operating at present is as follows: After carefully removing all decay (if any exists) from the external cavity, I cut directly into the nerve cavity, making as large an opening as is necessary for convenience; then with suitable instruments, remove the decomposed matter from the canal, together with the discolored bone which, in many instances, is found at the most depending portion of the pulp cavity. In some cases, however, of long standing, particularly where the teeth are soft and delicate, to remove all this discolored portion would render the crown of the tooth so weak as to endanger it; an allowance must be made for these cases. It is also important that the softened dentine, found near the apex of the fang, should be removed. This done, the canal must be washed throughout its whole extent with pure water, using for this purpose a small but powerful syringe, having the tube so bent as to allow of its introduction in the nerve cavity. The cavity should then be dried by filling to the apex of fang, or fangs, with floss silk, taking as fine a strand for each fang as can be successfully managed with the instrument, and leaving, for convenience of removal, a portion of each strand protruding from the cavity to the length of an inch or more. This done, I slightly moisten some strands of the silk with creosote, and then removing the first strands, I immediately refill with the latter, and then close the external opening with wax. The amount of creosote required, and the length of time I allow it to remain, depends much upon the time it has existed and the amount of decomposition to be overcome, but more upon the effect produced by the remedial agent itself—some of the apparently worst cases having been quickly cured, while others comparatively simple have required a course of treatment that has extended over weeks.

I usually examine these cases every twenty or forty-eight hours, renewing the floss silk *with creosote*, if it seems necessary, and without it when the first application seems to have been effectual. If after discontinuing the creosote for twenty-four or forty-eight hours, no trace of the odor of decomposition can be detected, I fill the fangs at once to the apex with gold, leaving the crown to be filled at a subsequent period. I make this delay in completing the operation with the view of avoiding the danger of periosteal inflammation, which might be the result of too much pressure at so critical a juncture.

In many recent cases of abscess it is only necessary to make one application of creosote, allowing it to remain twenty-four hours. In some cases, however, I have been obliged to renew it ten or twelve times.

The discharge usually ceases before the creosote has had its full effect. I have known it to cease, and after the removal of the creosote and the substitution of the dry floss, to recommence. Of course the creosote should be renewed under these circumstances.

After the dry floss has remained unchanged in the tooth for twenty-four hours, and without any unpromising symptoms having been noticed, I usually consider it safe to fill the nerve canal with gold, though where I have had trouble, or expect it, I frequently wait a day or two, sometimes a week or even more, being careful to keep the cavity well filled with dry floss and closely sealed with wax.

The external opening of the abscess does not disappear until from one to four weeks after the operation of filling with gold has been performed. I have been obliged, in some very obstinate cases, to lay the abscess open, and to apply alternately creosote and nitrate of silver.

Once I had reason to believe that periosteal inflammation had been excited by an excess of creosote, some of it having probably been forced through the foramen while packing in the floss which had been wet with it; the soreness resulting remained for a long time, but finally disappeared, and the operation was finished without any serious consequences.

Of course there are numerous cases of abscess which are beyond the reach of this remedy. Among these cases may be enumerated, abscess resulting from secondary hemorrhage, following the extirpation of the nervous pulp, and occurring after the *canal* has been filled with gold. The impossibility of removing the gold, puts these cases beyond the reach of the remedy in question, and the only chance will be in the use of nitrate of silver and creosote applied externally. Fortunately, accidents of this nature will seldom occur, provided sufficient precautions are taken previous to filling the roots.

Abscess occurring from periosteal inflammation, caused by external violence, is also beyond the reach of this remedy, particularly if accompanied by fracture of the alveolus. In these cases, the removal of the tooth and the fractured bones is usually necessary. Sometimes external violence may cause the death of the nerve, without serious injury to the socket or its lining membrane; when this occurs, and an abscess is formed, it can be successfully treated by the method described.

There is a diseased condition of the second and third inferior molars, which would result in abscess, were it not for the exceedingly dense structure of the bone in which these teeth are situated. The cause may be the same as those described above, but it results usually in a thickening of the periosteum, and in a deposit of bone in the socket of the tooth, commencing at the bottom of the socket, if the tooth has no antagonist, or laterally and more externally if it has. In the last case it can be noticed in the form of a hard broad tumor, with a base often greater than the width of the tooth. It is to be detected by passing the finger over the buccal surface of the jaw, at a point about one-third the length of the fang above its apex.

The above method of treatment would be successful in these cases previous to the formation of the tumor, but after it has once formed the extraction of the tooth becomes necessary, after which the disease gradually disappears.

As for the general success of this mode of treating alveolar abscess, I can only say that thus far (nearly four years having elapsed since I commenced it), the failures to cure abscess connected with the teeth of the upper jaw do not amount to five per cent. of the number treated. In the lower jaw I meet with more difficulty, and the failures are more frequent; recent cases, however, are almost invariably cured. In fact, a newly-formed abscess in either jaw may be considered a very tractable malady when this remedy can be applied. An abscess of long standing frequently is productive of serious injury to the socket and lining membrane of the tooth. The difficulty is also increased where there are a number of abscesses in the same jaw, particularly if they are connected with adjoining teeth; still I have succeeded in effecting a cure where there have been two, three, and in one case six abscesses adjoining.

NEW-YORK, 139 Fourth Avenue.

